# Supplementary material for: A community approach to mortality prediction in sepsis via gene expression analysis
Source: Nat Commun. 2018 Feb 15;9:694. doi: 10.1038/s41467-018-03078-2 (PMC5814463; doi:10.1038/s41467-018-03078-2)
Supplement: Supplementary file 1 — Supplementary Information [file 41467_2018_3078_MOESM1_ESM.pdf]

Supplementary Figures

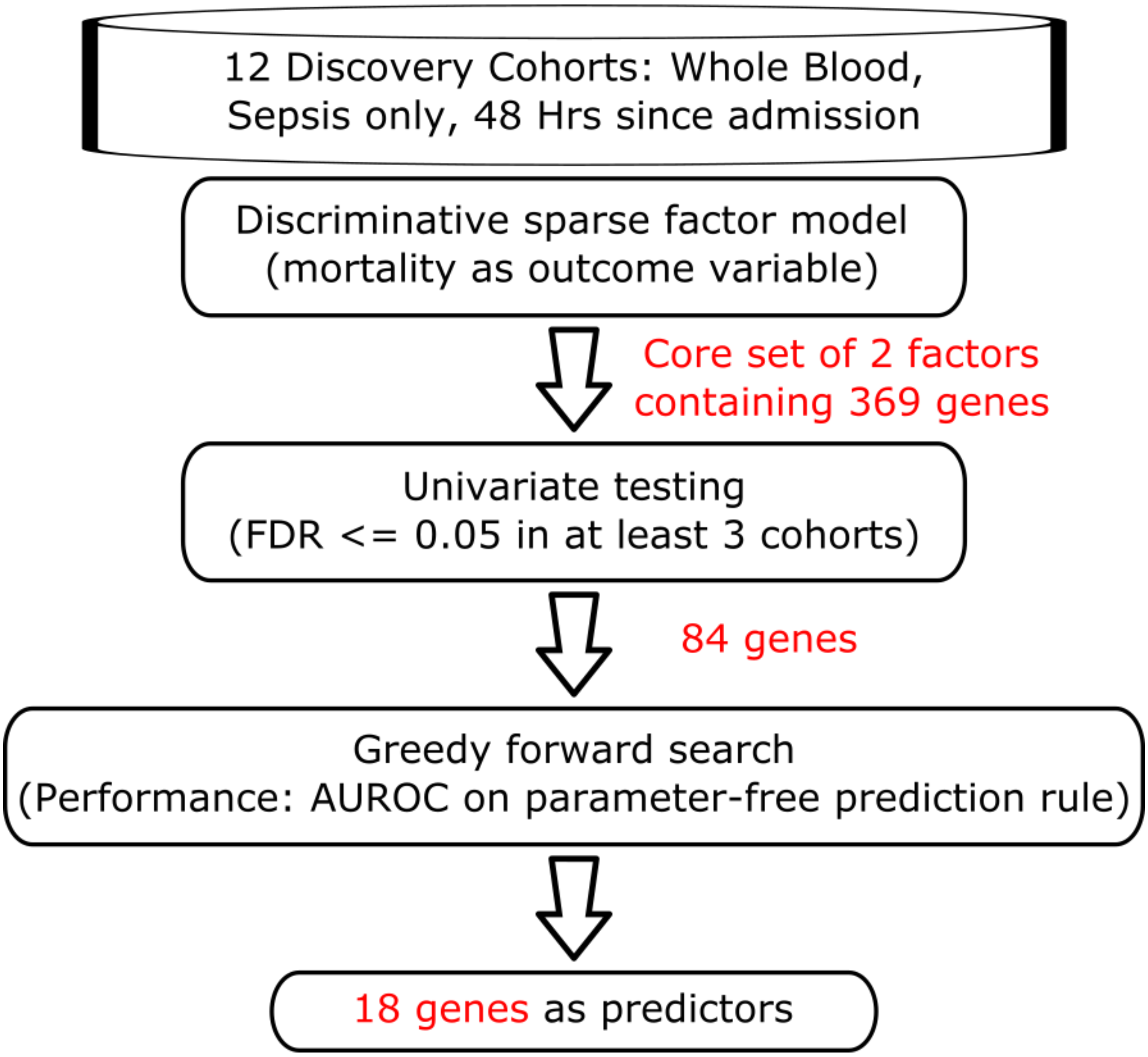

Supplementary Figure 1: Schematic of workflow of discovery of Duke model.

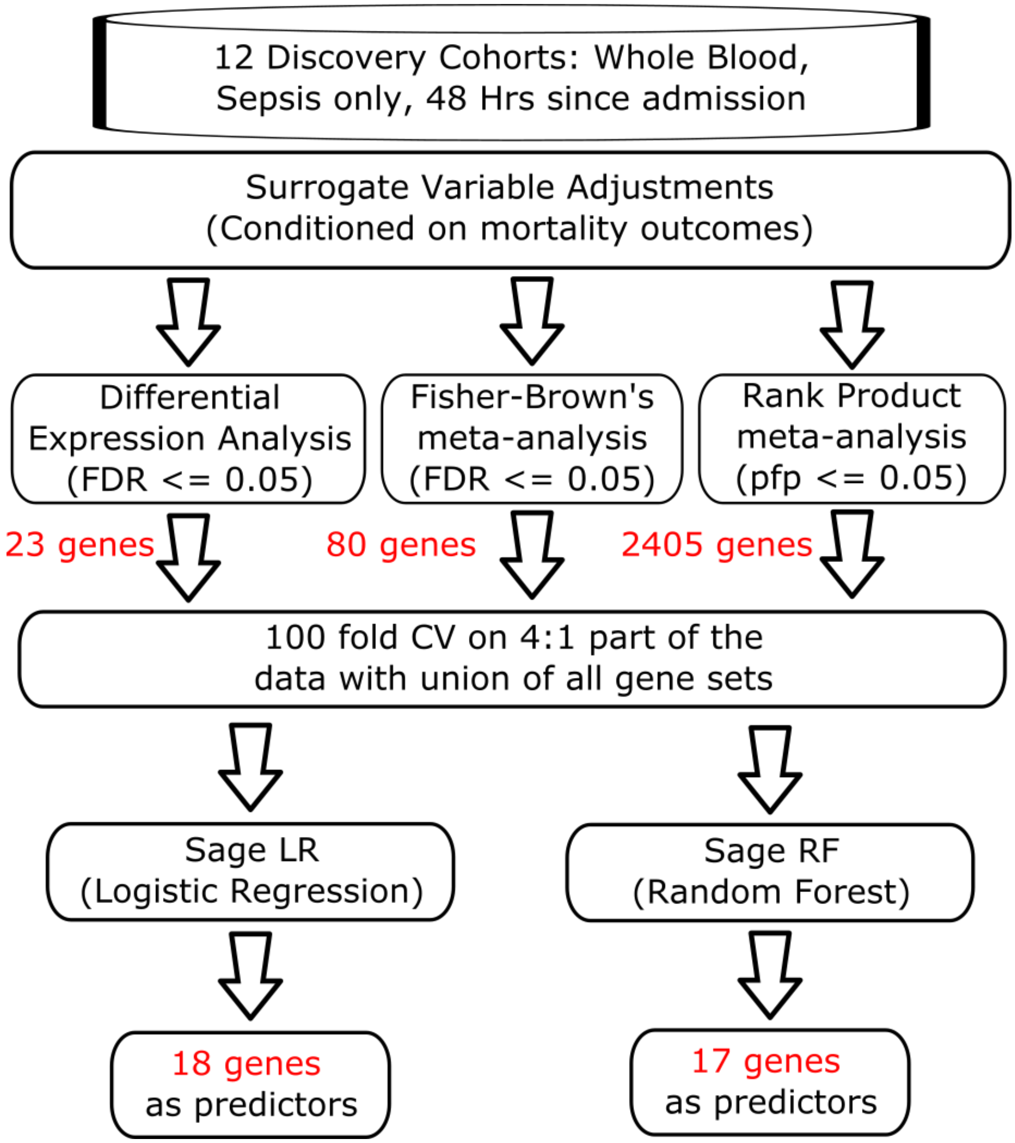

Supplementary Figure 2: Schematic workflow of discovery of Sage LR and Sage RF models.

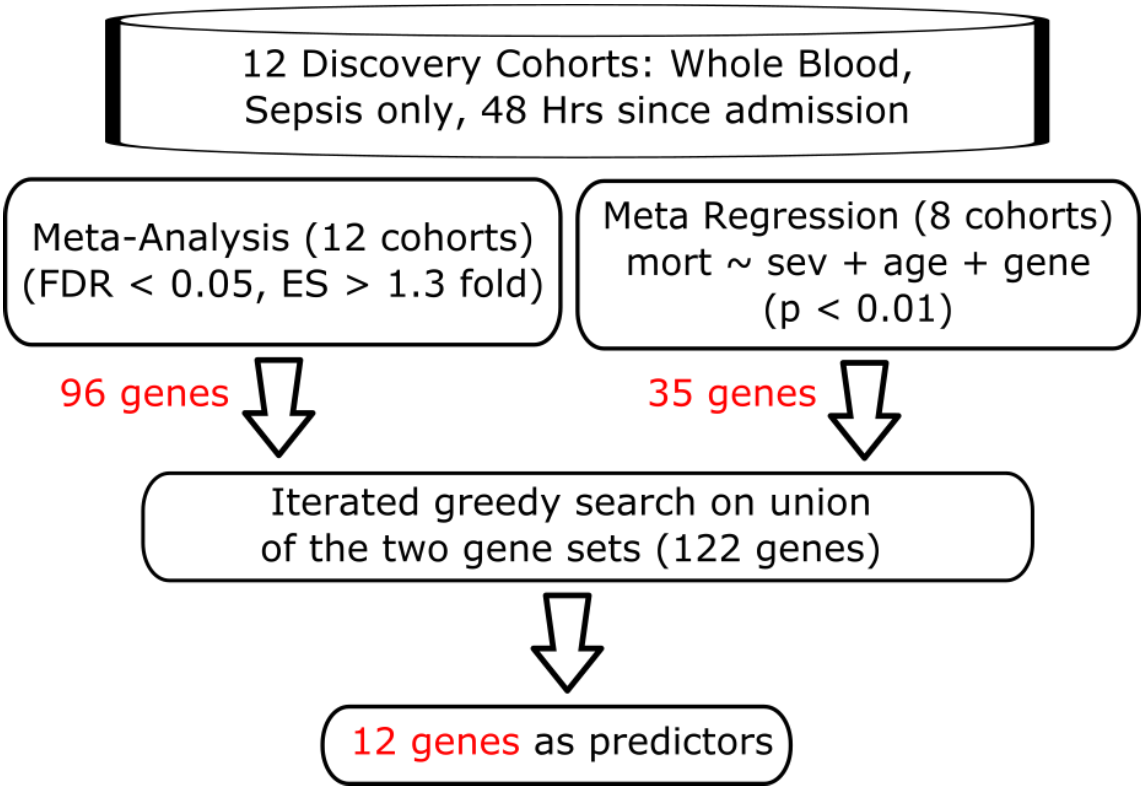

Supplementary Figure 3: Schematic of workflow of discovery of Stanford model.

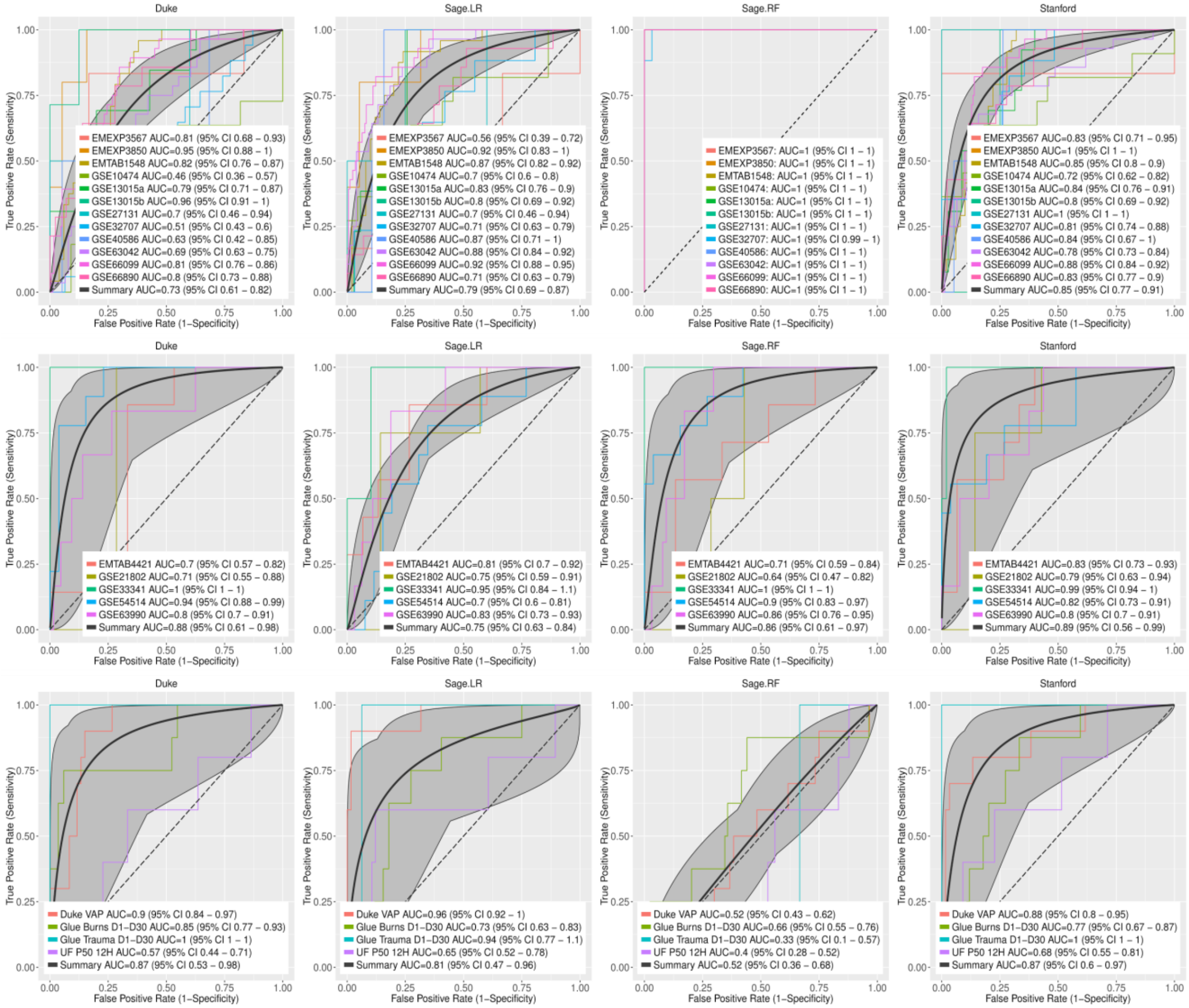

Supplementary Figure 4: Model performance showing individual ROC curves and summary ROC curve with confidence intervals (black and grey).

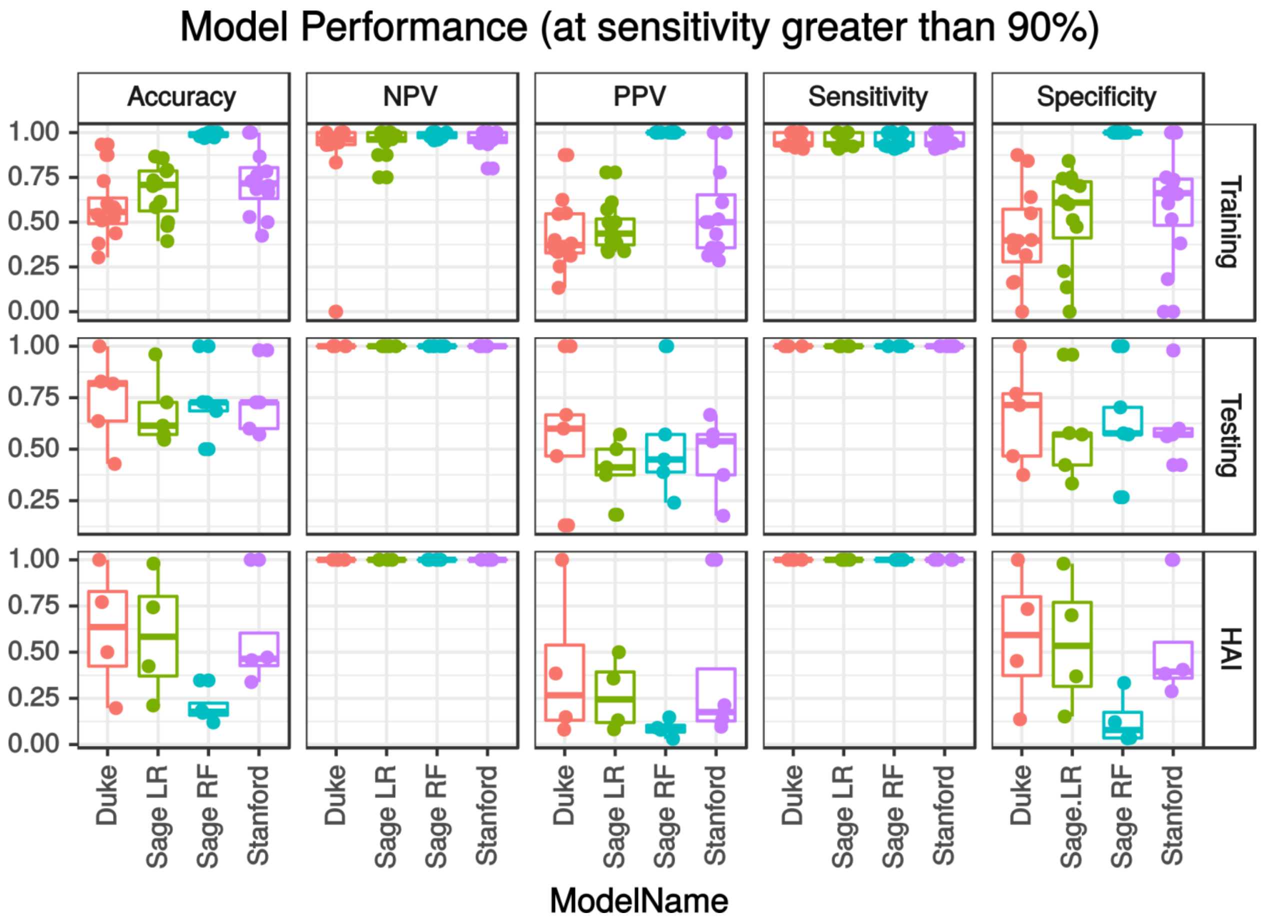

Supplementary Figure 5: Boxplots of other performance metrics for each model in individual datasets, with cutoffs set to the sensitivity nearest to 90%.

A.

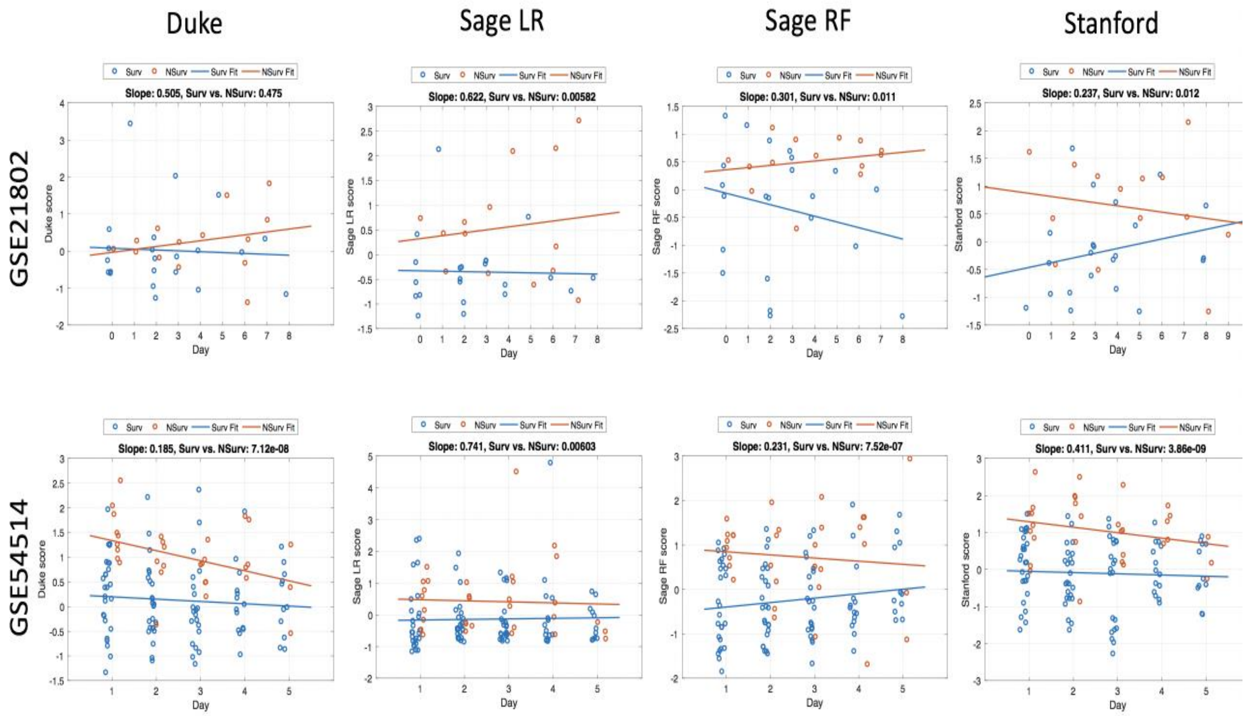

B.

Dataset	Model	Difference of slopes p-value	Difference of groups p-value
GSE21802	Duke	0.5053	0.475
	Sage LR	0.6216	**0.005**
	Sage RF	0.3007	**0.010**
	Stanford	0.2372	**0.012**
GSE54514	Duke	0.1846	**7.1×10^{-8}**
	Sage LR	0.7405	**0.006**
	Sage RF	0.2309	**7.5×10^{-7}**
	Stanford	0.4107	**3.8×10^{-9}**

Supplementary Figure 6: Longitudinal analysis of gene expression severity scores. Two datasets (GSE21802 and GSE54514) had available longitudinal samples from patients with sepsis over several days of hospital stay. There were no significant differences in slope (score over time) between the survivor and non-survivor groups. However, the scores remained significantly higher over time in non-survivors than in survivors in all but one comparison.

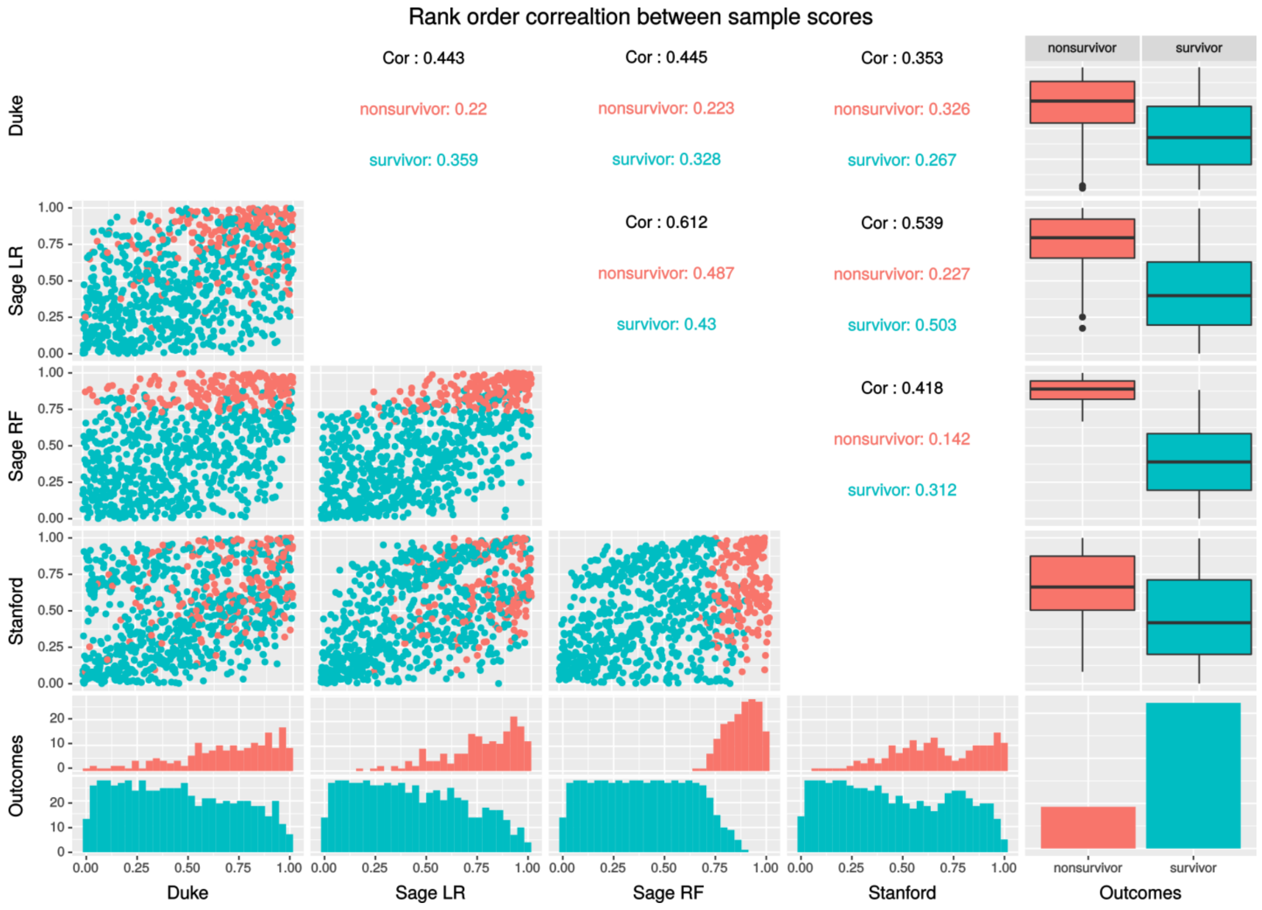

Supplementary Figure 7: Rank order correlation between sample scores across the four models for all samples.

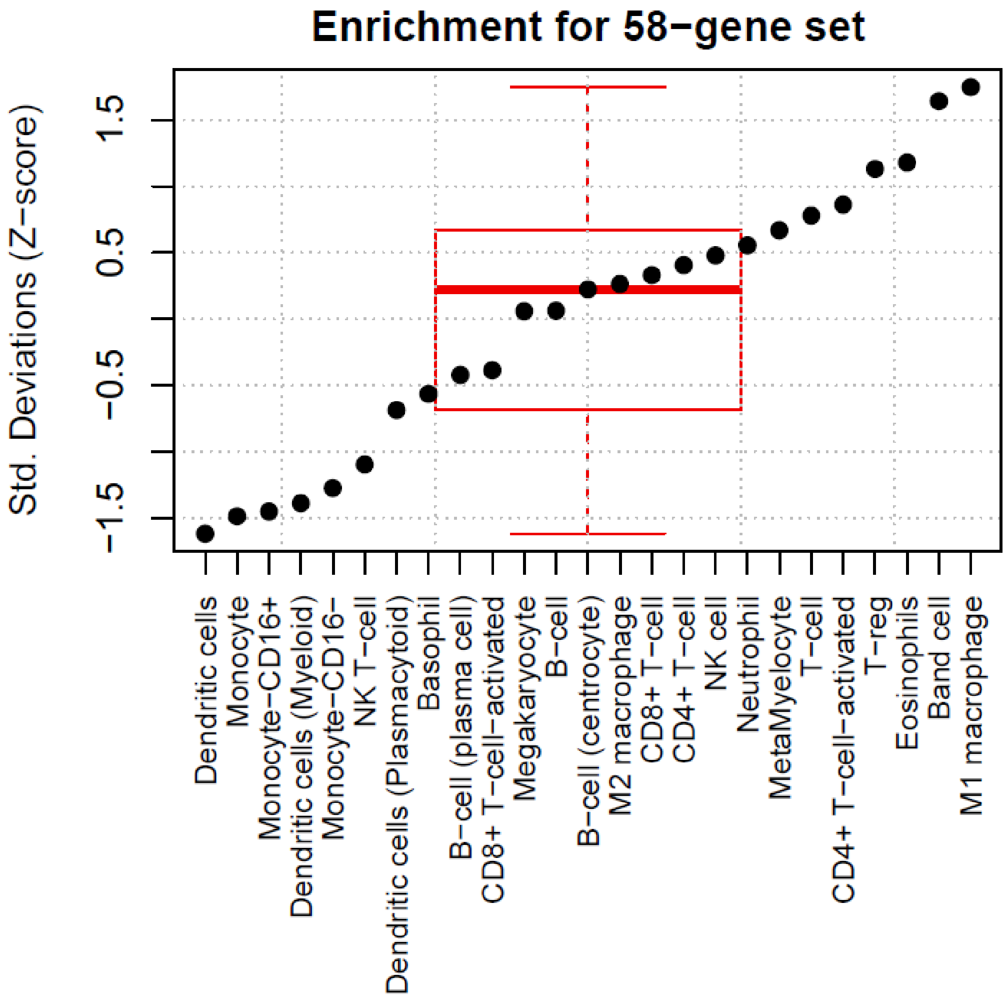

Supplementary Figure 8: Cell type enrichments of the entire set of 58 genes used across all four prediction models.

Supplementary Tables

Supplementary Table 1: Summary AUROCs for genomic models

Category	Parameter	Duke	Sage LR	Sage RF	Stanford
**Discovery**	**Summary**	0.73	0.79	1.0	0.85
	**95% CI**	0.62-0.82	0.69-0.87	1.0-1.0	0.77-0.91
	**Range**	0.46-0.96	0.69-0.87	1.0-1.0	0.72-1.0
**Validation**	**Summary**	0.88	0.76	0.87	0.89
	**95% CI**	0.62-0.98	0.64-0.86	0.61-0.97	0.57-0.98
	**Range**	0.70-1.0	0.70-0.95	0.64-1.0	0.79-0.99
**HAI**	**Summary**	0.87	0.81	0.53	0.87
	**95% CI**	0.62-0.98	0.64-0.86	0.61-0.97	0.57-0.98
	**Range**	0.70-1.0	0.70-0.95	0.64-1.0	0.79-0.99

Supplementary Table 2: Individual AUROCs for genomic models

Dataset	Duke	Sage LR	Sage RF	Stanford
**Discovery datasets**				
**E-MEXP-3567**	0.806	0.556	1.000	0.833
**E-MEXP-3850**	0.947	0.916	1.000	1.000
**E-MTAB-1548**	0.818	0.867	1.000	0.847
**GSE10474**	0.463	0.698	1.000	0.719
**GSE13015a**	0.787	0.831	1.000	0.835
**GSE13015b**	0.964	0.804	1.000	0.804
**GSE27131**	0.700	0.700	1.000	1.000
**GSE32707**	0.514	0.712	0.996	0.810
**GSE40586**	0.632	0.868	1.000	0.842
**GSE63042**	0.689	0.879	1.000	0.784
**GSE66099**	0.806	0.916	1.000	0.881
**GSE66890**	0.802	0.711	1.000	0.834
**Validation datasets**				
**E-MTAB-4421**	0.695	0.810	0.714	0.829
**GSE21802**	0.714	0.750	0.643	0.786
**GSE33341**	1.000	0.949	1.000	0.990

**GSE54514**	0.936	0.701	0.902	0.816
**GSE63990**	0.802	0.833	0.859	0.805
**HAI datasets**				
**Duke HAI**	0.905	0.963	0.522	0.875
**Glue Burns D1-D30**	0.850	0.731	0.656	0.769
**Glue Trauma D1-D30**	1.000	0.938	0.333	1.000
**UF P50 12H**	0.573	0.652	0.400	0.682

Supplementary Table 3: Comparison to genes previously associated with mortality. We found a total of 119 over-expressed and 1,164 under-expressed unique genes previously associated with mortality, which we assessed for prognostic accuracy in the validation datasets. We then compared the results to the output from the four models using paired t-tests.

Dataset	AUROC of combined 1,273 genes
**EMTAB4421**	0.581
**GSE21802**	0.679
**GSE 33341**	0.969
**GSE 54514**	0.761
**GSE 63990**	0.68
**Duke HAI**	0.83
**Glue Burns D1-D30**	0.417
**Glue Trauma D1-D30**	0.958
**UF P50 12H**	0.624

	Duke	Sage LR	Sage RF	Stanford
**mean difference**	0.108	0.092	-0.052	0.117
**P value**	0.046	0.059	0.595	0.014

Supplementary Table 4: Ensemble model performance characteristics

Dataset	AUROC (NS)	AUPR (NS)	PPV (NS)	NPV (NS)	PPV (S)	NPV (S)
**Discovery datasets**						
**EMEXP3567**	0.667	0.606	1.000	0.667	0.545	1.000
**EMEXP3850**	0.937	0.685	1.000	0.950	0.783	0.000
**EMTAB1548**	0.899	0.684	1.000	0.685	0.671	0.000
**GSE10474**	0.711	0.577	1.000	0.733	0.690	0.500
**GSE13015a**	0.881	0.624	1.000	0.778	0.723	0.000
**GSE13015b**	0.804	0.623	0.778	1.000	1.000	0.500
**GSE27131**	1.000	0.500	1.000	1.000	0.667	0.000
**GSE32707**	0.788	0.578	0.455	1.000	1.000	0.357
**GSE40586**	0.842	0.098	1.000	0.950	0.900	0.000
**GSE63042**	0.892	0.731	1.000	0.776	0.728	0.000
**GSE66099**	0.924	0.687	1.000	0.886	0.859	0.000
**GSE66890**	0.862	0.542	1.000	0.782	0.750	0.000
**Average**	**0.851**	**0.578**	**0.936**	**0.851**	**0.776**	**0.196**
**Std.Dev**	**0.095**	**0.165**	**0.165**	**0.127**	**0.139**	**0.325**
**Validation datasets**						
**EMTAB4421**	0.743	0.523	0.800	0.824	1.000	0.333
**GSE21802**	0.786	0.519	0.571	1.000	1.000	0.400

**GSE33341**	1.000	0.500	1.000	1.000	0.960	0.000
**GSE54514**	0.791	0.420	0.474	1.000	1.000	0.281
**GSE63990**	0.714	0.180	1.000	0.928	0.917	0.100
**Average**	**0.807**	**0.428**	**0.769**	**0.950**	**0.975**	**0.223**
**Std.Dev**	**0.113**	**0.145**	**0.242**	**0.077**	**0.037**	**0.167**

Supplementary Table 5: AUPR for genomic models (Individual datasets)

Dataset	Duke	Sage LR	Sage RF	Stanford
**Discovery datasets**				
**E-MEXP-3567**	0.686	0.530	0.833	0.746
**E-MEXP-3850**	0.616	0.475	0.800	0.800
**E-MTAB-1548**	0.558	0.637	0.958	0.620
**GSE10474**	0.321	0.525	0.909	0.594
**GSE13015a**	0.568	0.502	0.923	0.535
**GSE13015b**	0.816	0.600	0.857	0.623
**GSE27131**	0.163	0.208	0.500	0.500
**GSE32707**	0.333	0.533	0.938	0.658
**GSE40586**	0.176	0.225	0.500	0.238
**GSE63042**	0.378	0.670	0.964	0.555
**GSE66099**	0.374	0.662	0.964	0.468
**GSE66890**	0.541	0.408	0.929	0.597
**Validation datasets**				
**E-MTAB-4421**	0.350	0.540	0.407	0.519
**GSE21802**	0.442	0.519	0.392	0.519
**GSE33341**	0.500	0.208	0.500	0.292

**GSE54514**	0.694	0.372	0.713	0.613
**GSE63990**	0.246	0.204	0.240	0.182
**HAI datasets**				
**Duke HAI**	0.514	0.804	0.145	0.545
**Glue Burns D1-D30**	0.491	0.205	0.144	0.172
**Glue Trauma D1-D30**	0.000	0.250	0.015	0.000
**UF P50 12H**	0.085	0.157	0.054	0.129

Supplementary Table 6: Test characteristics at a high-sensitivity and a high-specificity cutoff. Shown are mean +/- sd of test characteristics across datasets within the three testing groups (discovery, validation, and HAI), for each of the four gene models, plus the baseline model (where all patients are judged all to be either survivors or nonsurvivors). All datasets are included.

Model	Data sets	True positive (percent)	False negative (percent)	False Positive (percent)	True negative (percent)	Sensitivity	Specificity	Accuracy
**High-sensitivity cutoff (sensitivity chosen near 95%)**								
Duke	Discovery	0.27 +/- 0.11	0.02 +/- 0.02	0.4 +/- 0.18	0.3 +/- 0.19	0.94 +/- 0.05	0.43 +/- 0.27	0.58 +/- 0.18
Sage.LR		0.27 +/- 0.11	0.02 +/- 0.02	0.32 +/- 0.16	0.39 +/- 0.23	0.94 +/- 0.05	0.53 +/- 0.28	0.66 +/- 0.15
Sage.RF		0.27 +/- 0.11	0.02 +/- 0.02	0 +/- 0.01	0.71 +/- 0.12	0.94 +/- 0.05	1 +/- 0.01	0.98 +/- 0.02
Stanford		0.27 +/- 0.11	0.02 +/- 0.02	0.27 +/- 0.18	0.44 +/- 0.23	0.94 +/- 0.05	0.6 +/- 0.3	0.72 +/- 0.18
Baseline model (all nonsurvivors)		0.29 +/- 0.12	0 +/- 0	0.71 +/- 0.12	0 +/- 0	1 +/- 0	0 +/- 0	0.29 +/- 0.12
Duke	Validation	0.2 +/- 0.13	0.01 +/- 0.02	0.22 +/- 0.21	0.57 +/- 0.24	0.95 +/- 0.07	0.72 +/- 0.23	0.77 +/- 0.21
Sage.LR		0.2 +/- 0.13	0.01 +/- 0.02	0.29 +/- 0.14	0.5 +/- 0.23	0.95 +/- 0.07	0.61 +/- 0.2	0.69 +/- 0.14
Sage.RF		0.2 +/- 0.13	0.01 +/- 0.02	0.22 +/- 0.14	0.57 +/- 0.26	0.95 +/- 0.07	0.69 +/- 0.2	0.76 +/- 0.15
Stanford		0.2 +/- 0.13	0.01 +/- 0.02	0.27 +/- 0.16	0.52 +/- 0.25	0.95 +/- 0.07	0.64 +/- 0.21	0.72 +/- 0.17
Baseline model (all nonsurvivors)		0.21 +/- 0.14	0 +/- 0	0.79 +/- 0.14	0 +/- 0	1 +/- 0	0 +/- 0	0.21 +/- 0.14
Duke	HAI	0.07 +/- 0.04	0.01 +/- 0.01	0.35 +/- 0.36	0.57 +/- 0.37	0.94 +/- 0.07	0.62 +/- 0.39	0.64 +/- 0.36
Sage.LR		0.07 +/- 0.04	0.01 +/- 0.01	0.32 +/- 0.38	0.6 +/- 0.37	0.94 +/- 0.07	0.66 +/- 0.41	0.67 +/- 0.37
Sage.RF		0.07 +/- 0.04	0.01 +/- 0.01	0.63 +/- 0.17	0.29 +/- 0.17	0.94 +/- 0.07	0.32 +/- 0.18	0.36 +/- 0.17
Stanford		0.07 +/- 0.04	0.01 +/- 0.01	0.32 +/- 0.27	0.6 +/- 0.29	0.94 +/- 0.07	0.64 +/- 0.29	0.67 +/- 0.27
Baseline model (all nonsurvivors)		0.08 +/- 0.05	0 +/- 0	0.92 +/- 0.05	0 +/- 0	1 +/- 0	0 +/- 0	0.08 +/- 0.05
**High-specificity cutoff (specificity chosen near 95%)**								
Duke	Discovery	0.11 +/- 0.1	0.18 +/- 0.1	0.03 +/- 0.02	0.68 +/- 0.1	0.37 +/- 0.25	0.96 +/- 0.02	0.79 +/- 0.1
Sage.LR		0.1 +/- 0.06	0.19 +/- 0.1	0.04 +/- 0.02	0.67 +/- 0.11	0.37 +/- 0.22	0.95 +/- 0.03	0.78 +/- 0.1
Sage.RF		0.29 +/- 0.12	0 +/- 0	0.03 +/- 0.02	0.68 +/- 0.1	1 +/- 0	0.96 +/- 0.02	0.97 +/- 0.02
Stanford		0.15 +/- 0.11	0.14 +/- 0.1	0.04 +/- 0.02	0.67 +/- 0.11	0.51 +/- 0.28	0.95 +/- 0.03	0.82 +/- 0.11
Baseline model (all survivors)		0 +/- 0	0.29 +/- 0.12	0 +/- 0	0.71 +/- 0.12	0 +/- 0	1 +/- 0	0.71 +/- 0.12
Duke	Validation	0.06 +/- 0.08	0.15 +/- 0.16	0.05 +/- 0.02	0.74 +/- 0.16	0.45 +/- 0.42	0.93 +/- 0.04	0.8 +/- 0.18
Sage.LR		0.09 +/- 0.12	0.12 +/- 0.09	0.05 +/- 0.02	0.74 +/- 0.16	0.37 +/- 0.29	0.93 +/- 0.04	0.83 +/- 0.09

Sage.RF		0.05 +/- 0.07	0.16 +/- 0.15	0.05 +/- 0.02	0.74 +/- 0.16	0.4 +/- 0.42	0.93 +/- 0.04	0.79 +/- 0.17
Stanford		0.13 +/- 0.11	0.08 +/- 0.05	0.05 +/- 0.02	0.74 +/- 0.16	0.61 +/- 0.31	0.93 +/- 0.04	0.87 +/- 0.06
Baseline model (all survivors)		0 +/- 0	0.21 +/- 0.14	0 +/- 0	0.79 +/- 0.14	0 +/- 0	1 +/- 0	0.79 +/- 0.14
Duke	HAI	0.03 +/- 0.02	0.05 +/- 0.04	0.04 +/- 0	0.88 +/- 0.05	0.48 +/- 0.43	0.95 +/- 0	0.91 +/- 0.04
Sage.LR		0.04 +/- 0.06	0.04 +/- 0.03	0.04 +/- 0	0.88 +/- 0.05	0.28 +/- 0.43	0.95 +/- 0	0.91 +/- 0.03
Sage.RF		0.01 +/- 0.01	0.07 +/- 0.05	0.04 +/- 0	0.88 +/- 0.05	0.06 +/- 0.12	0.95 +/- 0	0.88 +/- 0.05
Stanford		0.04 +/- 0.04	0.04 +/- 0.03	0.04 +/- 0	0.88 +/- 0.05	0.51 +/- 0.42	0.95 +/- 0	0.91 +/- 0.03
Baseline model (all survivors)		0 +/- 0	0.08 +/- 0.05	0 +/- 0	0.92 +/- 0.05	0 +/- 0	1 +/- 0	0.92 +/- 0.05

Supplementary Table 7: Test characteristics at a high-sensitivity and a high-specificity cutoff in combination with clinical severity score. Shown are mean +/- sd of test characteristics across datasets within the three testing groups (discovery, validation, and HAI), for each of the four gene models, plus the baseline model (where all patients are judged to be either all survivors or all nonsurvivors). Only those datasets with available subject-level clinical data (Supplementary Table 5) are included.

**Model**	**Datasets**	**True positive (%)**	**False negative (%)**	**False Positive (%)**	**True negative (%)**	**Sens-itivity**	**Spec-ificity**	**Acc-uracy**	**True positive (%)**	**False negative (%)**	**False Positive (%)**	**True negative (%)**	**Sens-itivity**	**Spec-ificity**	**Acc-uracy**
**High-sensitivity cutoff (sensitivity chosen near 95%)**		**Gene score or severity score (separately)**							**Joint severity score + gene score model**						
Severity only	Discovery	0.26 +/- 0.07	0.01 +/- 0.01	0.36 +/- 0.24	0.37 +/- 0.26	0.95 +/- 0.03	0.5 +/- 0.34	0.63 +/- 0.25	N/A						
Duke		0.25 +/- 0.07	0.02 +/- 0.01	0.41 +/- 0.15	0.32 +/- 0.2	0.94 +/- 0.04	0.43 +/- 0.25	0.58 +/- 0.16	0.25 +/- 0.07	0.02 +/- 0.01	0.3 +/- 0.22	0.43 +/- 0.26	0.94 +/- 0.04	0.57 +/- 0.34	0.68 +/- 0.23
Sage.LR		0.25 +/- 0.07	0.02 +/- 0.01	0.35 +/- 0.15	0.38 +/- 0.21	0.94 +/- 0.04	0.51 +/- 0.25	0.64 +/- 0.15	0.25 +/- 0.07	0.02 +/- 0.01	0.26 +/- 0.21	0.47 +/- 0.26	0.94 +/- 0.04	0.63 +/- 0.33	0.73 +/- 0.22
Sage.RF		0.25 +/- 0.07	0.02 +/- 0.01	0 +/- 0.01	0.73 +/- 0.08	0.94 +/- 0.04	1 +/- 0.01	0.98 +/- 0.02	0.25 +/- 0.07	0.02 +/- 0.01	0 +/- 0	0.73 +/- 0.08	0.94 +/- 0.04	1 +/- 0	0.98 +/- 0.01
Stanford		0.25 +/- 0.07	0.02 +/- 0.01	0.26 +/- 0.19	0.47 +/- 0.22	0.94 +/- 0.04	0.64 +/- 0.27	0.73 +/- 0.2	0.25 +/- 0.07	0.02 +/- 0.01	0.24 +/- 0.17	0.49 +/- 0.21	0.94 +/- 0.04	0.65 +/- 0.26	0.74 +/- 0.18
Severity only	Validation	0.3 +/- 0.09	0.02 +/- 0.02	0.41 +/- 0.04	0.27 +/- 0.09	0.92 +/- 0.08	0.39 +/- 0.1	0.57 +/- 0.06	N/A						
Duke		0.3 +/- 0.09	0.02 +/- 0.02	0.25 +/- 0.14	0.43 +/- 0.22	0.92 +/- 0.08	0.62 +/- 0.26	0.73 +/- 0.13	0.3 +/- 0.09	0.02 +/- 0.02	0.26 +/- 0.18	0.42 +/- 0.25	0.92 +/- 0.08	0.6 +/- 0.3	0.72 +/- 0.17
Sage.LR		0.3 +/- 0.09	0.02 +/- 0.02	0.34 +/- 0.13	0.34 +/- 0.15	0.92 +/- 0.08	0.5 +/- 0.21	0.64 +/- 0.12	0.3 +/- 0.09	0.02 +/- 0.02	0.4 +/- 0.03	0.28 +/- 0.07	0.92 +/- 0.08	0.41 +/- 0.07	0.58 +/- 0.03
Sage.RF		0.3 +/- 0.09	0.02 +/- 0.02	0.29 +/- 0.08	0.39 +/- 0.14	0.92 +/- 0.08	0.57 +/- 0.14	0.69 +/- 0.09	0.3 +/- 0.09	0.02 +/- 0.02	0.27 +/- 0.11	0.41 +/- 0.17	0.92 +/- 0.08	0.59 +/- 0.19	0.71 +/- 0.12
Stanford		0.3 +/- 0.09	0.02 +/- 0.02	0.32 +/- 0.1	0.36 +/- 0.09	0.92 +/- 0.08	0.53 +/- 0.12	0.66 +/- 0.1	0.3 +/- 0.09	0.02 +/- 0.02	0.32 +/- 0.07	0.35 +/- 0.05	0.92 +/- 0.08	0.52 +/- 0.07	0.65 +/- 0.07
Severity only	HAI	0.05 +/- 0.03	0 +/- 0	0.38 +/- 0.39	0.57 +/- 0.41	1 +/- 0	0.6 +/- 0.43	0.62 +/- 0.39	N/A						
Duke		0.05 +/- 0.03	0 +/- 0	0.43 +/- 0.41	0.51 +/- 0.43	1 +/- 0	0.53 +/- 0.44	0.57 +/- 0.41	0.05 +/- 0.03	0 +/- 0	0.21 +/- 0.2	0.74 +/- 0.23	1 +/- 0	0.78 +/- 0.22	0.79 +/- 0.2
Sage.LR		0.05 +/- 0.03	0 +/- 0	0.53 +/- 0.41	0.42 +/- 0.44	1 +/- 0	0.44 +/- 0.44	0.47 +/- 0.41	0.05 +/- 0.03	0 +/- 0	0.27 +/- 0.29	0.68 +/- 0.31	1 +/- 0	0.71 +/- 0.31	0.73 +/- 0.29
Sage.RF		0.05 +/- 0.03	0 +/- 0	0.66 +/- 0.29	0.29 +/- 0.32	1 +/- 0	0.3 +/- 0.33	0.34 +/- 0.29	0.05 +/- 0.03	0 +/- 0	0.36 +/- 0.47	0.58 +/- 0.49	1 +/- 0	0.61 +/- 0.51	0.64 +/- 0.47
Stanford		0.05 +/- 0.03	0 +/- 0	0.41 +/- 0.36	0.54 +/- 0.38	1 +/- 0	0.56 +/- 0.38	0.59 +/- 0.36	0.05 +/- 0.03	0 +/- 0	0.22 +/- 0.23	0.73 +/- 0.25	1 +/- 0	0.77 +/- 0.24	0.78 +/- 0.23
**Model**	**Datasets**	**True positive (%)**	**False negative (%)**	**False Positive (%)**	**True negative (%)**	**Sens-itivity**	**Spec-ificity**	**Acc-uracy**	**True positive (%)**	**False negative (%)**	**False Positive (%)**	**True negative (%)**	**Sens-itivity**	**Spec-ificity**	**Acc-uracy**

**<u>High-specificity cutoff</u> (specificity chosen near 95%)**		**Gene score or severity score (separately)**							**Joint severity score + gene score model**						
Severity only	Discovery	0.11 +/- 0.09	0.16 +/- 0.12	0.05 +/- 0.02	0.68 +/- 0.08	0.43 +/- 0.36	0.93 +/- 0.03	0.79 +/- 0.13	N/A						
Duke		0.09 +/- 0.06	0.18 +/- 0.1	0.04 +/- 0.02	0.69 +/- 0.07	0.35 +/- 0.25	0.94 +/- 0.03	0.77 +/- 0.1	0.13 +/- 0.08	0.15 +/- 0.12	0.04 +/- 0.02	0.69 +/- 0.07	0.5 +/- 0.34	0.94 +/- 0.03	0.81 +/- 0.12
Sage.LR		0.12 +/- 0.04	0.15 +/- 0.08	0.04 +/- 0.02	0.69 +/- 0.07	0.46 +/- 0.19	0.94 +/- 0.03	0.8 +/- 0.08	0.16 +/- 0.07	0.11 +/- 0.1	0.04 +/- 0.02	0.69 +/- 0.07	0.61 +/- 0.29	0.94 +/- 0.03	0.84 +/- 0.11
Sage.RF		0.27 +/- 0.08	0 +/- 0	0.04 +/- 0.02	0.69 +/- 0.07	1 +/- 0	0.94 +/- 0.03	0.96 +/- 0.02	0.27 +/- 0.08	0 +/- 0	0.04 +/- 0.02	0.69 +/- 0.07	1 +/- 0	0.94 +/- 0.03	0.96 +/- 0.02
Stanford		0.14 +/- 0.08	0.13 +/- 0.09	0.04 +/- 0.02	0.69 +/- 0.07	0.52 +/- 0.3	0.94 +/- 0.03	0.83 +/- 0.1	0.16 +/- 0.07	0.11 +/- 0.08	0.04 +/- 0.02	0.69 +/- 0.07	0.6 +/- 0.25	0.94 +/- 0.03	0.85 +/- 0.09
Severity only	Validation	0.15 +/- 0.04	0.17 +/- 0.03	0.03 +/- 0.03	0.64 +/- 0.04	0.46 +/- 0.04	0.95 +/- 0.04	0.79 +/- 0.02	N/A						
Duke		0.08 +/- 0.1	0.24 +/- 0.17	0.07 +/- 0.03	0.61 +/- 0.1	0.31 +/- 0.41	0.9 +/- 0.06	0.69 +/- 0.19	0.24 +/- 0.06	0.09 +/- 0.05	0.03 +/- 0.03	0.64 +/- 0.04	0.74 +/- 0.16	0.95 +/- 0.04	0.88 +/- 0.05
Sage.LR		0.15 +/- 0.14	0.17 +/- 0.07	0.07 +/- 0.03	0.61 +/- 0.1	0.43 +/- 0.32	0.9 +/- 0.06	0.76 +/- 0.04	0.16 +/- 0.06	0.17 +/- 0.03	0.03 +/- 0.03	0.64 +/- 0.04	0.47 +/- 0.12	0.95 +/- 0.04	0.8 +/- 0.02
Sage.RF		0.07 +/- 0.09	0.25 +/- 0.16	0.07 +/- 0.03	0.61 +/- 0.1	0.27 +/- 0.35	0.9 +/- 0.06	0.68 +/- 0.18	0.19 +/- 0.01	0.13 +/- 0.07	0.03 +/- 0.03	0.64 +/- 0.04	0.62 +/- 0.14	0.95 +/- 0.04	0.83 +/- 0.05
Stanford		0.21 +/- 0.08	0.12 +/- 0.02	0.07 +/- 0.03	0.61 +/- 0.1	0.63 +/- 0.11	0.9 +/- 0.06	0.82 +/- 0.01	0.16 +/- 0.04	0.17 +/- 0.05	0.03 +/- 0.03	0.64 +/- 0.04	0.5 +/- 0.06	0.95 +/- 0.04	0.8 +/- 0.03
Severity only	HAI	0.03 +/- 0.03	0.02 +/- 0.01	0.2 +/- 0.21	0.75 +/- 0.23	0.43 +/- 0.4	0.79 +/- 0.23	0.78 +/- 0.22	N/A						
Duke		0.03 +/- 0.02	0.03 +/- 0.03	0.07 +/- 0.01	0.88 +/- 0.03	0.62 +/- 0.4	0.93 +/- 0.01	0.91 +/- 0.03	0.04 +/- 0.02	0.01 +/- 0.01	0.07 +/- 0.01	0.88 +/- 0.03	0.82 +/- 0.17	0.93 +/- 0.01	0.92 +/- 0.02
Sage.LR		0.01 +/- 0.01	0.04 +/- 0.04	0.07 +/- 0.01	0.88 +/- 0.03	0.4 +/- 0.53	0.93 +/- 0.01	0.89 +/- 0.04	0.03 +/- 0.03	0.03 +/- 0.04	0.07 +/- 0.01	0.88 +/- 0.03	0.6 +/- 0.53	0.93 +/- 0.01	0.9 +/- 0.04
Sage.RF		0 +/- 0.01	0.05 +/- 0.03	0.07 +/- 0.01	0.88 +/- 0.03	0.06 +/- 0.1	0.93 +/- 0.01	0.88 +/- 0.03	0.03 +/- 0.02	0.02 +/- 0.03	0.07 +/- 0.01	0.88 +/- 0.03	0.66 +/- 0.43	0.93 +/- 0.01	0.91 +/- 0.03
Stanford		0.02 +/- 0	0.04 +/- 0.03	0.07 +/- 0.01	0.88 +/- 0.03	0.46 +/- 0.47	0.93 +/- 0.01	0.89 +/- 0.04	0.03 +/- 0.02	0.02 +/- 0.03	0.07 +/- 0.01	0.88 +/- 0.03	0.66 +/- 0.43	0.93 +/- 0.01	0.91 +/- 0.03

Supplementary Table 8. Agreement between models. Classification labels were obtained from study-wise thresholds corresponding to 90% sensitivity (non-survivors). Consensus corresponds to patients correctly classified by at least 3 of 4 models, whereas no consensus represents correct classifications by 1 or 2 models.

Dataset	Always misclassified	No Consensus	Consensus
**Discovery datasets**			
**GSE40586**	0.0952	0.5238	0.3810
**GSE10474**	0.5152	0.1818	0.3030
**GSE13015a**	0.0417	0.3542	0.6042
**GSE13015b**	0.0000	0.2000	0.8000
**GSE27131**	0.0000	0.4286	0.5714
**GSE32707**	0.0625	0.4792	0.4583
**GSE63042**	0.1923	0.4615	0.3462
**GSE66099**	0.0854	0.2915	0.6231
**GSE66890**	0.2456	0.2456	0.5088
**EMTAB1548**	0.1216	0.1622	0.7162
**EMEXP3567**	0.0000	0.2500	0.7500
**EMEXP3850**	0.0000	0.1250	0.8750
**Discovery Average**	**11.33+/-14.94**	**30.86+/-13.86**	**57.81+/-18.49**
**Validation datasets**			
**GSE54514**	0.0857	0.2571	0.6571
**EMTAB4421**	0.0909	0.2727	0.6364
**GSE21802**	0.1818	0.0909	0.7273
**GSE33341**	0.0000	0.0000	1.0000
**GSE63990**	0.0429	0.2286	0.7286
**Validation Average**	**8.03+/-6.76**	**16.99+/-11.91**	**74.99+/-15.58**

HAI datasets			
**UF P50 12H**	0.2394	0.4930	0.2676
**Glue Trauma D1-D30**	0.0000	0.0000	1.0000
**Glue Burns D1-D30**	0.1087	0.3696	0.5217
**Duke HAI**	0.0000	0.3000	0.7000
**HAI Average**	**8.70+/-11.38**	**29.06+/-20.95**	**62.23+/-30.80**
**Total Average**	**10.04+/-12.39**	**27.22+/-15.23**	**62.74+/-20.62**

Supplementary Table 9: Genomic features of sepsis mortality (union of all models)

Direction	Predictors
**Up-regulated in mortality (31 genes)**	*DEFA4, CD163, PER1, RGS1, HIF1A, SEPP1, C11orf74, CIT, CFD, DDIT4, IFI27, IL1R2, IL8, MAFF, OCLN, B4GALT4, BPI, CD24, CEP55, CTSG, G0S2, MPO, MT1G, NDUFV2, PAM, PSMA6, TRIB1, CKS2, MKI67, POLD3, PLK1*
**Down-regulated in mortality (27 genes)**	*LY86, TST, OR52R1, KCNJ2, AIM2, APH1A, CCR2, EIF5A, GSTM1, HIST1H3H, NT5E, RAB40B, VNN3, ABCB4, CTSS, IKZF2, TGFBI, CST3, CBFA2T3, RCBTB2, CX3CR1, CD5, MTMR11, CLEC10A, EMR3, DHRS7B, CEACAM8*

Supplementary Table 10: Nominally enriched pathways. Significance was set at a p value ≤ 0.05 and gene sets were only included with at least 3 genes overlapping. **FC, fold change. FDR, false discovery rate.**

**Supplementary Table 12 (a): Fisher's Exact Test**		
**Gene Set Name**	**Odds Ratio**	**FDR**
Positive regulation of T cell activation	2.12E+01	2.32E-02
Positive regulation of T cell proliferation	3.78E+01	2.14E-02
Process. cytokine-mediated signaling pathway	1.40E+01	3.00E-02
RHO GTPases activate CIT	1.69E+02	5.16E-03
**Supplementary Table 12 (b): Gene Set Enrichment Analysis**		
**Enriched in Non-Survivors**		
**Gene Set Name**	**Log-FC**	**FDR**
Amoebiasis	4.82E-01	0.00E+00
MAPK signaling pathway	4.82E-01	0.00E+00
Interleukin-1 signaling	4.82E-01	0.00E+00
HTLV-I infection	3.11E-01	2.15E-264
Antigen processing: Ubiquitination & Proteasome degradation	3.54E-01	1.52E-237
Positive regulation of cell cycle process	3.76E-01	8.00E-199
Regulation of cellular ketone metabolic process	3.76E-01	8.00E-199
TCF dependent signaling in response to WNT	3.76E-01	8.00E-199
Interferon-gamma-mediated signaling pathway	3.49E-01	2.45E-195
Inflammatory bowel disease (IBD)	3.49E-01	2.45E-195
Viral myocarditis	3.49E-01	2.45E-195
Graft-versus-host disease	3.49E-01	2.45E-195
Antigen processing and presentation	3.49E-01	2.45E-195
Staphylococcus aureus infection	3.49E-01	2.45E-195
Influenza A	3.49E-01	2.45E-195
Epstein-Barr virus infection	3.49E-01	2.45E-195

Enriched in Survivors		
**GeneSetName**	**Log-FC**	**FDR**
Dendrite development	-3.61E-01	5.58E-261
Regulation of mitosis	-3.61E-01	5.58E-261
Generation of neurons	-3.61E-01	5.58E-261
Golgi organization	-3.61E-01	5.58E-261
Neurogenesis	-3.61E-01	5.58E-261
Transmembrane transport of small molecules	-4.06E-01	5.22E-246
Metabolism of lipids and lipoproteins	-4.06E-01	5.22E-246
PPARA activates gene expression	-4.06E-01	5.22E-246
ATP catabolic process	-3.24E-01	5.86E-217
Mitotic nuclear division	-3.01E-01	1.20E-159
Immunoregulatory interactions between a Lymphoid and a non-Lymphoid cell	-3.67E-01	6.96E-137
Adenylate cyclase-inhibiting G-protein coupled receptor signaling pathway	-3.49E-01	1.20E-129
Negative regulation of inflammatory response	-3.50E-01	5.99E-120

Supplementary Notes

HAI Dataset Descriptions

Glue Grant (Burns & Trauma) Study: The Inflammation and Host Response to Injury Program (Glue Grant) whole blood / buffy coat cohorts[1] were treated as previously described[2]. The Glue Grant datasets contain two cohorts: patients admitted with severe trauma, and patients admitted with severe burns. The trauma cohorts further include two sub-cohorts, one which sampled buffy coat, and the other which sampled sorted cells; the sorted-cells cohort were excluded from further study. Trauma patients were sampled at the following days after admission: 0.5, 1, 4, 7, 14, 21, 28 days; Burn patients were sampled at admission, and then at the time of their burn operations. The Glue Grant patients were classified as 'infected' if they had a nosocomial infection (pneumonia, urinary tract infection, catheter-related bloodstream infection, etc.), a surgical infection (excluding superficial wound infections), or underwent surgery for perforated viscus. In burn patients, burn wound cultures of <100 CFU/g were not considered as infections. Only patients with samples drawn within ± 24 hours of the day of diagnosis of infection were included. The initial 24 hours after admission was not included, as the index admissions were not for infectious causes. All deaths within 30 days were scored as deaths, regardless of cause. Use of the Glue Grant was approved by both the Glue Grant Consortium and the Stanford University IRB (protocol 29798).

UF p50 Study: This prospective observational study was performed between January 2012 and August 2016 at UF Health Shands Hospital, and was reviewed and approved by the Institutional Review Board (IRB) prior to initiation. Patients currently in the SICU or recently transferred to the SICU suspected of having early sepsis (within 48 hours of admission), and requiring initiation of a sepsis management protocol, were screened for inclusion in the study. Adults (age ≥ 18 years) with sepsis, severe sepsis, or septic shock as defined by the 2001 SCCM/ESICM/ACCP/ATS/SIS International Sepsis Definitions

Conference at index admission (and with no prior entry into the sepsis protocol at UF Health during that admission) were included. Patients were excluded if any of the following were present: (1) Expected lifespan less than 3 months due to severe pre-existing comorbidities (e.g. recurrent, advanced, or metastatic cancer); (2) Severe traumatic brain injury, defined as evidence of neurologic injury on CT scan and a GCS < 8 after resuscitation; (3) Refractory shock with anticipated death within 12 hours; (4) Uncontrollable sepsis due to an inability to achieve source control (e.g. irreversible disease states such as unresectable necrotic bowel); (5) Patient or patient's family not committed to aggressive management of the patient's condition; (6) Severe CHF (NYHA Class IV); (7) Child-Pugh Class C liver disease or pre-liver transplant; (8) Known HIV with CD4 count <200 cells/mm^3; (9) Organ transplant recipients on immunosuppressive agents or patients receiving chronic corticosteroids or immunosuppressive agents for other reasons; (10) Pregnancy; (11) Incarceration; (12) Institutionalized patients; (13) Inability to obtain informed consent; (14) Chemotherapy or radiotherapy within 30 days prior to onset of sepsis; or (15) Spinal cord injury resulting in permanent sensory and/or motor deficits. Data were obtained from the Sepsis and Critically Illness Research Center (SCIRC) at the University of Florida College of Medicine. Supported in part by P50 GM111152, from NIGMS, SCIRC is conducting a five year observational study on the incidence of chronic critical illness in patients with sepsis and their long-term physical and functional outcomes.

Duke Hospital-Acquired Infection (HAI) Study: This prospective, multi-center, observational cohort study enrolled patients ≥ 18 years of age hospitalized within the medical or surgical wards, intensive care units, or step-down units of participating medical centers at Duke University Health System, Duke Regional Hospital, Durham Veterans

Affairs Medical Center, and the University of North Carolina–Chapel Hill Hospital System. The purpose of the study was to understand the clinic–molecular risk factors and manifestations of HAI, inclusive of ventilator–associated pneumonia (VAP) and non–VAP HAI. Serial samples were obtained including pre– and post–sepsis onset. For the purposes of this analysis, we focused only on the time point corresponding to sepsis onset, as determined by a clinical adjudication process.

Supplementary Methods

Prognostic Model Analysis Descriptions

Duke University:

We propose a two-step process for identifying signatures of mortality in patients with sepsis. As seen in the Supplementary Figure 1, the first step consists of a discriminative factor model[3] that attempts to jointly estimate the covariance structure of the data from a low-rank representation consisting of sparse factors, while also producing a sparse predictive model of mortality based on the latent factor scores also estimated by the model. The model has a clear interpretation by virtue of its sparseness property, each factor defines a subset of genes and the predictive model identifies which factors are discriminative (associated) with mortality. In addition, since the model captures the covariance structure of the data, factors not associated with mortality can often be found to be associated with other large sources of variation such as batch effects and/or demographic features. One known disadvantage of sparse factor models is that although it produces sparse factors, the size of the factors is usually in the hundreds of genes, which is less than ideal in applications were translation to targeted platforms admittedly require small gene signatures.

The second step of our methodology consists of down-selecting from the subset(s) of genes deemed by the factor model as discriminative of mortality, we call this collection of genes our core set. To this end, we perform univariate testing (1-way ANOVA) on each of the genes in the core set, individually for each discovery set to better quantify within-cohort mortality associations. Next, we filter-out genes not statistically significant in a proportion of the discovery sets (25% or 3 studies in the experiments) to then optimize the gene signature by greedy forward search on the remaining genes while sorting them by maximum raw p-value across discovery datasets. The best signature is one such that the weighted average AUC is maximum. The prediction rule of our final predictive model is parameter-free and it is defined as the geometric mean of the up-regulated genes minus the geometric mean of the down-regulated genes in the original scale of the data, i.e., prior log-transformation. Note that this prediction rule is used during the greedy search but is not part of the sparse predictive model of our factor model. We opted for a parameter-free prediction rule as opposed to a parametric model, e.g., logistic regression, to simplify the final model and to make it less dependent on the scale of the data.

We applied this method to identify gene signatures associated with mortality in patients

with sepsis. The model estimated 16 factors from which only two were statistically significant with respect to survival status at FDR < 0.05. This discriminative factor consisted of 369 genes that form our signature core set. In order to obtain a smaller signature and a parameter-free classification model, we performed univariate testing on each one of the 12 discovery sets while restricting genes to our core set. We discarded genes that were not statistically significant at the p < 0.05 level in at least 3 discovery sets (84 of 369). Next we optimized the gene signature by greedy search on the remaining 84 genes sorted by raw p-value across datasets and using AUC as the performance metric. The greedy algorithm resulted in a final 18 gene set down-selected from the original 84 core set, from which 6 were up-regulated in non-survivors (CEACAM8, TRIB1, CKS2, MKI67, POLD3 and PLK1), while 12 were down-regulated in non-survivors (TGFBI, LY86, CST3, CBFA2T3, RCBTB2, TST, CX3CR1, CD5, MTMR11, CLEC10A, EMR3 and DHRS7B).

Sage LR and RF:

Data Adjustments: For the purpose of selecting features that are relevant to mortality alone, we adjusted each cohort using a surrogate variable analysis (SVA)[4] conditioned on mortality status. This step avoids feature sets that could be confounded with other known and unknown covariates such as gender, age, severity and batch effects. Therefore, for each cohort, for each gene, we fit a regression model with mortality (known covariate) and surrogate variables (unknown covariates). The resulting residuals of the model is added back to the mortality coefficients and used for all downstream predictions.

Feature Reduction: Machine learning algorithms tend to perform better with reduced feature space[5]. Therefore, SVA adjusted data sets with 9340 genes expressed in all the 12 different discovery/training datasets is reduced to a smaller feature set using three different methodologies. (i) First method fits a regression model for every gene in each cohort with mortality (as a dependent variable) and the resulting coefficients were tested for differential expression between survivors and nonsurvivors. This method results in 23 differentially expressed genes in all the 12 discovery datasets at an FDR of 0.05. This approach, considering a maximum p-value of a gene in all studies, is a stringent criterion for selection. (ii) The second approach combines differential expression p-values for each gene in every cohort using Fisher's chi-squared statistics with a Brown's correction[6] for non-independence/correlated effects

between different datasets. This approach is moderately conservative and results in 80 genes for prediction at an FDR of 0.05. (iii) The third approach is a rank product[7] methodology were each gene in a given sample were relatively ranked according to their expression values and the ranks across samples were combined using a rank product. The significance of the detection is assessed by a non-parametric permutation test. At an FDR of 0.05 this method results in 2405 genes across 12 different discovery datasets. Finally, we took union of most expressed genes from all three methods resulting in 2367 unique genes from the 9340 as significant features for our multi-cohort analysis.

Model training: SVA adjusted gene expression of 2367 genes in 12 different datasets were used to train a penalized logistic regression (Sage LR)[8] and probability random forest (Sage RF)[9] models to predict nonsurvivors of sepsis from survivors. Discovery set were split into 100 different partitions of 80%-20% of training data and only the 80% of training data was used to train the models. Coefficients or variable importance scores for every gene in each model is relatively ranked and combined across all 100 splits to obtain a final ranking. 897 and 327 genes were considered as predictors in at least one of the 100 different Sage LR or Sage RF models, respectively.

Model pruning: All selected features from the 100 models may or may not be relevant. Therefore, as a final feature selection process, we pruned the above models based on the relative ranking of coefficients obtained from 100 different models and using a BIC criteria[10], which penalizes for increased model complexity. In the end, we obtain 9 up and 9 down regulated genes in Sage LR and 13 up and 4 down regulated genes in Sage RF models as predictors of mortality.

Sage LR: SVA adjusted data sets were used to infer gene signatures associated with mortality. 9340 genes that were commonly expressed in all the 12 different discovery datasets were reduced to a smaller feature set using the above mentioned three methodologies. A generalised linear model with penalized maximum likelihood calculated via coordinate descent methodology[11] was used to reduce genomic features from the selected 2367 genes. This resulted in a 18 gene model for predicting mortality in non-survivors at a summary AUROC of 0.79, 0.76 and 0.81 in the discovery, validation and HAI datasets, respectively. These 18 genes include 9 up-regulated (CFD, DDIT4, DEFA4, IFI27, IL1R2, IL8, MAFF, OCLN, RGS1) and 9 down-regulated (AIM2, APH1A, CCR2, EIF5A, GSTM1, HIST1H3H, NT5E, RAB40B, VNN3) in nonsurvivors.

Sage RF: Like the Sage LR model, the Sage RF model used 2367 significantly differentially expressed genes which were at least selected by one of the method as features for our multi-cohort analysis. Sage RF model used penalized classification probability-based random forest (classRF) algorithm as described in Malley et al[12]. In brief, the classRF algorithm first creates a bootstrap sample set with replacement from the available samples by leaving out certain percentage of training data. Later a classification tree is built for each bootstrap to the greatest extent possible, but requiring at least a minimum of 10% of the samples as nodes. Finally, the probability of each sample is calculated as the proportion of predicted non-survivors in the final nodes of all the bootstraps. To reduce the set of features that predict mortality, penalised classRF is used. In general, sage RF model displayed near perfect prediction in all the discovery data, with a summary AUROC of 1. However, the performance decreased in the validation data sets and shown significantly reduced performance in the HAI sets. This model resulted in an imbalanced 17 gene set with 13 (B4GALT4, BPI, CD24, CEP55, CTSG, DDIT4, G0S2, MPO, MT1G, NDUFV2, PAM, PSMA6, SEPP1) of them up-regulated in non-survivors and 4 (ABCB4, CTSS, IKZF2, NT5E) down-regulated in non-survivors.

Stanford University:

We applied two analytic methods to discover genes significantly associated with mortality (Supplementary Figure 3). After selecting the input datasets, we first combined effect sizes within datasets using Hedges' g[13], and then evaluated summary effects with a DerSimonian-Laird meta-analysis[14]. Significance thresholds were set at a false discovery rate (FDR) of 0.05, with a summary effect size greater than 1.3 fold (in non-log space).

We next performed a meta-regression analysis in the datasets which supplied phenotype data of clinical severity and age. For each cohort, for each gene, the model was a regression on mortality (dependent) as a function of clinical severity plus age plus gene expression level. To keep the scales between datasets similar solely for the regression analysis, (1) all clinical severity scores were converted to log-odds mortality, based on models in their describing papers, and (2) all datasets were ComBat-normalized[4] together prior to meta-analysis (this method resets the location and scale of each gene, but within-cohort differences are preserved). The meta-regression was carried out using the closed-form method-of-moments random-effects model variation[15] of the synthesis-of-slopes regression method described by Becker and Wu (2007)[16].

Thus, in this case, a gene was considered to be significant if it had statistically conserved regression coefficients (betas) across all datasets for the prediction of mortality independent of clinical severity and age. An uncorrected p value < 0.01 was deemed significant.

In the final step of the analysis, we took as significant the union of the gene sets deemed to be significant both by standard multi-cohort analysis and by meta-regression. These genes were then used in a greedy iterated search model, where a greedy forward search was allowed to run to completion, followed by a greedy backward search, and then another greedy forward search. This method iterated until it reached a stable gene set. This algorithm is designed to find maxima closer to the global maximum than a simple forward search. Only the discovery datasets were used in the search, each dataset was evaluated separately, and the functions maximized the weighted AUC, which is the sum of the AUC of each discovery dataset multiplied by its sample size.

In the greedy search, and with the final gene set, the gene score is defined as the geometric mean of the gene expression level for all positive genes minus the geometric mean of the gene expression level of all negative genes multiplied by the ratio of counts of positive to negative genes. This was calculated for each sample in a dataset separately. Genes not present in an entire dataset were excluded; genes missing for individual samples were set to one.

The initial multi-cohort meta-analysis for differential gene expression between survivors and nonsurvivors at admission yielded 96 genes significant at FDR<0.05 and effect size >1.3-fold. In the regression analysis there were 35 genes significant at p<0.01. Notably, the top three most-significant genes in the meta-regression were all from the same pathway, namely, neutrophil azurophilic granules: *DEFA4*, *CTSG*, and *MPO*. The union of the meta-analysis and meta-regression gene sets was 122 genes, which we took as our 'significant' gene list to feed into the iterated greedy search. The algorithm ran to completion, producing a 12-gene set. The genes upregulated in patients with mortality were: DEFA4, CD163, PER1, RGS1, HIF1A, SEPP1, C11orf74, and CIT, and the downregulated genes were: LY86, TST, OR52R1, and KCNJ2.

Ensemble Model:

The aim of the ensemble model is to aggregate the classifications submitted by the individual four models, by effectively leveraging the consensus as well as diversity among these predictions. We performed a stacking-based penalized SVM, a heterogeneous ensemble

methodology. This method learns a meta-classifier (second level predictor) with the prediction scores from the four base classifiers. In order to reduce the over-fitting of the ensemble classifier, the training set for classifications were generated through a leave-one-cohort-out cross-validation procedure applied to all the discovery datasets. To address the potential calibration issue, we also investigated two different normalization procedures; z-score based (mean=0, SD=1) and rank based scaling (maximum=1, minimum=0), applied to the raw base classification scores. Normalized scores were then used to train a meta-classifier model. To this end, we used penalisedSVM[17] package in R with elastic SCAD penalty.

Comparison to Prior Published Gene Sets:

In order to contrast the present findings with prior published results, we searched for all papers that examined transcriptome-wide changes in sepsis associated with increasing severity or mortality[18–23]. In each of these papers, genes were classified as 'over-expressed' or 'under-expressed' in association with increasing severity. We took the union of all these differentially expressed genes as inputs, and took the difference of geometric means of these two sets to make a single score. We then measured the AUROC for prediction of mortality using this composite score, and compared to the present scores. To compare this level of performance to the four current models, we used matched t-tests, as well as calculating the mean difference in AUROCs. We did this in the validation cohorts only to prevent bias.

Supplementary References

1. Xiao, W. *et al.* A genomic storm in critically injured humans. *J. Exp. Med.* **208,** 2581–2590 (2011).
2. Sweeney, T. E., Shidham, A., Wong, H. R. & Khatri, P. A comprehensive time-course-based multicohort analysis of sepsis and sterile inflammation reveals a robust diagnostic gene set. *Sci. Transl. Med.* **7,** 287ra71 (2015).
3. Henao, R., Yuan, X. & Carin, L. Bayesian Nonlinear Support Vector Machines and Discriminative Factor Modeling. in *NIPS* (2014).
4. Leek, J. T., Johnson, W. E., Parker, H. S., Jaffe, A. E. & Storey, J. D. The sva package for removing batch effects and other unwanted variation in high-throughput experiments. *Bioinformatics* **28,** 882–883 (2012).
5. Fefferman, C., Charles, F., Sanjoy, M. & Hariharan, N. Testing the manifold hypothesis. *J. Amer. Math. Soc.* **29,** 983–1049 (2016).
6. Brown, M. B. 400: A Method for Combining Non-Independent, One-Sided Tests of Significance. *Biometrics* **31,** 987 (1975).
7. Hong, F. *et al.* RankProd: a bioconductor package for detecting differentially expressed genes in meta-analysis. *Bioinformatics* **22,** 2825–2827 (2006).
8. Friedman, J., Hastie, T. & Tibshirani, R. Regularization Paths for Generalized Linear Models via Coordinate Descent. *J. Stat. Softw.* **33,** 1–22 (2010).
9. Liaw, A. & Wiener, M. Classification and Regression by randomForest. *R News* **2,** 18–22 (2002).
10. Bayesian Information Criteria. in *Springer Series in Statistics* 211–237 (2008).
11. Friedman, J., Hastie, T. & Tibshirani, R. Regularization Paths for Generalized Linear Models via Coordinate Descent. *J. Stat. Softw.* **33,** 1–22 (2010).
12. Malley, J. D., Kruppa, J., Dasgupta, A., Malley, K. G. & Ziegler, A. Probability machines: consistent probability estimation using nonparametric learning machines. *Methods Inf. Med.* **51,** 74–81 (2012).
13. Hedges, L. V. Distribution Theory for Glass's Estimator of Effect Size and Related Estimators. *J.*

Educ. Behav. Stat. **6,** 107 (1981).

14. DerSimonian, R. & Laird, N. Meta-analysis in clinical trials. *Control. Clin. Trials* **7,** 177–188 (1986).
15. Chen, H., Manning, A. K. & Dupuis, J. A method of moments estimator for random effect multivariate meta-analysis. *Biometrics* **68,** 1278–1284 (2012).
16. Becker, B. J. & Meng-Jia, W. The Synthesis of Regression Slopes in Meta-Analysis. *Stat. Sci.* **22,** 414–429 (2007).
17. Zhang, H. H., Ahn, J., Lin, X. & Park, C. Gene selection using support vector machines with non-convex penalty. *Bioinformatics* **22,** 88–95 (2006).
18. Pachot, A. *et al.* Systemic transcriptional analysis in survivor and non-survivor septic shock patients: a preliminary study. *Immunol. Lett.* **106,** 63–71 (2006).
19. Wong, H. R. *et al.* Genome-level expression profiles in pediatric septic shock indicate a role for altered zinc homeostasis in poor outcome. *Physiol. Genomics* **30,** 146–155 (2007).
20. Parnell, G. P. *et al.* Identifying key regulatory genes in the whole blood of septic patients to monitor underlying immune dysfunctions. *Shock* **40,** 166–174 (2013).
21. Almansa, R. *et al.* Transcriptomic correlates of organ failure extent in sepsis. *J. Infect.* **70,** 445–456 (2015).
22. Tsalik, E. L. *et al.* An integrated transcriptome and expressed variant analysis of sepsis survival and death. *Genome Med.* **6,** 111 (2014).
23. Bauer, P. R. *et al.* Diagnostic accuracy and clinical relevance of an inflammatory biomarker panel for sepsis in adult critically ill patients. *Diagn. Microbiol. Infect. Dis.* **84,** 175–180 (2016).
